# A phenomenological inquiry into farmers’ experiences growing cotton in Punjab, Pakistan

**DOI:** 10.1038/s41598-024-62950-y

**Published:** 2024-06-15

**Authors:** Saleem Ashraf, Khalid Mahmood Ch, Ijaz Ashraf, Nadeem Akbar

**Affiliations:** 1 Institute of Agricultural Extension, Education and Rural Development, University of Agriculture Faisalabad, Faisalabad, Pakistan; 2 Department of Agronomy, University of Agriculture Faisalabad, Faisalabad, Pakistan

**Keywords:** Lived experiences, Climate change, Variety, Attitude, Pesticides, Fertilizers, Whitefly, Pink Boll Worm, Plant sciences, Psychology

## Abstract

Sustainability in cotton production is inevitable because producing more cotton means more employment, economic acceleration, and industrial expansion. India, China, the United States, Brazil, and Pakistan contribute 74% of worldwide cotton production. Pakistan is contributing only 5%, despite the high potential of cotton. The average yield of cotton in Pakistan is stagnant at 570.99 kg hm^−2^, whereas it entails the highest cost of production among all other crops. The yield obtained in Pakistan is less than the potential, profitability is drastically lessening, and farmers are abandoning cotton for alternative kharif crops. Some traditional quantitative studies have unveiled different factors that affect cotton production. However, an in-depth qualitative study has never been conducted in Pakistan to explore the root causes of growing cotton crop failure. Following Moustakas’s traditional phenomenological guidelines, this phenomenological study was conducted in the district of Rahim Yar Khan in the core cotton zone of Punjab province. A total of 10 interviews were conducted with purposively selected cotton growers based on a criterion: (i) having more than 10 years of cotton growing experience, (ii) being a cotton grower, and (iii) having at least 10 years of formal schooling. Interviews were conducted face to face on an interview guide. One interview lasted 45–50 min, and responses were recorded and analyzed using a thematic analysis approach. A total of 6 themes emerged from the collected data, including (i) climate change, (ii) varietal problems, (iii) pesticide usage, (iv) sense of institutional services, (v) attitude of farmers and (vi) soil health and environment. These six merging themes contributed to cotton crop failure and yield decline. The deep exploration further summarized that researchers, extensionists, and farmers need to seriously consider variety, sowing time, and the environment to revive cotton crops. The detailed recommendations and policy guidelines are presented in this paper, highlighting the cotton sector’s research, development and investment areas.

## Introduction

Worldwide, *Gossypium hirsutum* L. (upland cotton) covers approximately 95% of the total area of 33–35 million hectares of cotton cultivation, constituting about 2.5% of arable land^1^. Cotton has an annual economic impact of around $600 billion worldwide, and it remains the leading natural fibre produced and traded globally^2,3^. The cotton industry encompasses the cotton industry, which is industrialized in approximately 150 countries and provides livelihoods for approximately 100 million families^4,5^. Global cotton production is expected to reach 141.3 million bales by the year 2031–32, with a projected increase in yield of 1.63 bales per acre during the same period. In 2022–23, the worldwide cotton trade amounted to 48.7 million bales, and this trade volume could rise to 53 million bales in 2031–32, driven by the growing demand for textiles in Bangladesh and Vietnam^6^.

The cotton industry employs approximately 250 million people worldwide, with nearly 7% of the labour force in developing countries involved in this field. Cotton has become the primary non-food agricultural commodity as the dominant natural fibre. The combined cotton production of five countries constitutes 74% of the total global output^7^. The cotton sector contributes significantly to the economies of developing countries. It employs an estimated 150 million people across 75 countries, making it an essential contributor to the 2030 Agenda for Sustainable Development^8^. China, India, Brazil, USA, Australia, Turkiye and Pakistan produced 5977, 5722, 3232, 2634, 1250, 1028 and 833 “000” tonnes of cotton lint in 2023^9^. Global cotton production is likely to grow at 1.6% annually, increasing from 126.5 million bales in 20222-23 to approximately 141.3 million bales in 2031–32. India, China, the United States, Brazil and Pakistan will retain their dominance in cotton production, sharing 23, 22, 14, 11 and 05% of global cotton production^6^.

Pakistan is the fifth-largest producer, contributing 5% to global cotton production^6^. The cotton crop is vital to Pakistan’s economy, particularly its agro-based industry, which employs approximately 50% of the industrial workforce. The cotton crop products chain also accounts for about 60% of the country’s total exports^10^. In Pakistan, cotton occupies a prominent role as the most crucial cash crop. Among the country’s farming community, it is often referred to as "white gold" due to its exceptional ability to generate substantial revenue for the farmers^11^. Nearly 1.7 million farmers throughout Pakistan cultivate cotton with the hope of sustaining their livelihoods^12^. Ideally, sustainable cotton production is essential in Pakistan to support the textile sector, maintain employment, support livelihoods, and earn capital for the country. For instance, Cottonseed oil currently fulfils 18.1% of Pakistan’s requirements for edible oil. By 2030, the total demand for this purpose will reach 5.5 million tonnes, of which 2 million tonnes will be supplied locally. Higher production of cotton can help the country meet this oil demand^13^.

The cotton-producing region of Pakistan spans approximately 1200 kms along the Indus River, ranging from latitudes 27°N to 35°N and altitudes between 27 and 155 m. The soil composition transitions from clay loam to sandy, with a prevalence of clay-rich soil in the southern areas^14^. Cotton cultivation in Pakistan mainly occurs in the Punjab and Sindh regions^15^ under the wheat-cotton cropping system, which is by far the second-largest cropping system in India and Pakistan, spanning over 4.19 Mha^16^. Climatic variations have more influence in Punjab^17^, and early sowing is suggested. Although cotton planting usually occurs from April to June, harvesting occurs between August and December. The late sowing of cotton, especially after 20 April, can cause a reduction in production^18^. Most cotton production, accounting for over 90%, is carried out by small-scale farmers cultivating less than five hectares of land. Over the years, the production of cotton has declined. The average yield stood at 753 kg per hectare in 2017, dropping to 445 kg per hectare by 2021^19^ and the average cotton yield in Pakistan is around 570.99 kg hm^−2^ was reported by Razzaq, Zafar^20^. The current cotton yield in Pakistan is considerably lower than its potential, and the cost of production is much higher compared to other cotton-producing countries. The average yield of cotton in Pakistan is about 570.99 kg.hm^-2^^20^. Whereas, worldwide, the average cotton lint yield is about 800 kg/ha, with irrigated conditions achieving 3500 kg/ha, and raingrown cotton production systems varying between 800 and 800 kg/ha^21^. As per the Data Book of ICAC^9^, the average yield of cotton in China is 1992 kg/ha, Brazil 1931 kg/ha, Australia 1877 kg/ha, Turkiye 1775 kg/ha and Mexico obtained 1560 kg/ha yield of cotton. The average yield in Pakistan was 388 kg/ha, which is significantly lower and makes this crop less profitable. Ch, Ashraf^22^ and Mubeen, Ahmad^23^ concluded that cotton production in Pakistan has declined due to environmental, climate change, water-related, agronomic, and socio-economic constraints. Moreover, Pakistan’s cotton industry has lost its competitiveness in the international perspective^24^.

**Figure 1:**
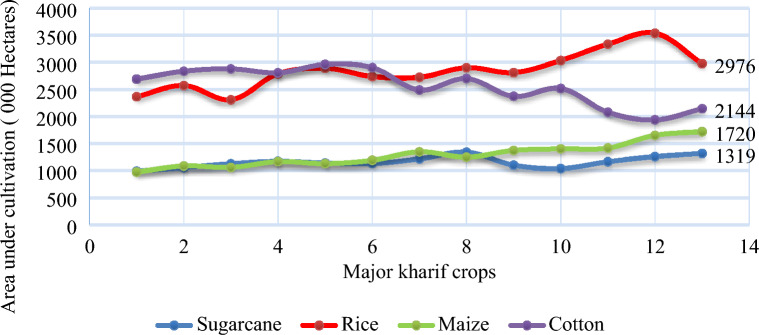
The area under cultivation of major crops from 2010 to 2023. This shows that other Kharif crops like Sugarcane, Rice, and Maize compete with cotton. As a result, the area under cotton cultivation is gradually decreasing, and the area of crops, significantly and sugarcane, is increasing at the expense of cotton. In thirteen years, the sugarcane area reached 1319 000 hectares from 988 000 Hectares, whereas the area of Maize increased from 974 to 1720 000 Hectares. This is a notion that cotton has become less profitable and competitive than kharif crops like sugarcane and maize. Source: Economic Surveys of Pakistan, 2023.

Due to an increasing lack of profitability, there has been a significant decline in cotton cultivation and overall production and yield^22^^,^^25^. Robinson^26^ observed a reduction in the cotton cultivation area, which he attributed to the expansion of sugarcane cultivation. Sugarcane has emerged as a formidable competitor to cotton due to its higher production yields and profitability. The area statistics from 2010 to 2023 are portrayed in Fig. 1 for reference.

The production dynamics of cotton have been extensively researched in Pakistan. Various disciplines, including genetics, sociology, and agricultural extension, have provided insights into cotton’s prospects and future outlook, particularly in the Punjab province, which contributes 80% of the country’s total cotton production. Government reports indicate a substantial 29% decrease in cotton production in 2015 and a 17% decline in both cotton area and production in 2019^27,28^. At the same time, some research studies anticipate an increase in cotton production^29,30^. Ashraf, Sangi^31^ projected a drastic decline in cotton production in Punjab province by 2025, attributing it to various factors related to agronomics, physical conditions, human factors, crop production practices, crop protection, and advisory services approaches. In another research study, Gul, Paul^32^ revealed that Pakistan’s cotton production and average yield will increase by 2025. Punjab’s cotton crop area will expand, but output and average yield continue to fall.

Climate changes, irregularities in rainfall patterns, temperature fluctuations during the cotton season causing physiological stress on crops, and an increase in weeds, insects, pests, and diseases have been identified as limiting factors by Raza and Ahmad^33^, Rashid, Husnain^34^, Abbas, Khan^35^, and Arshad, Raza^36^ The Government of Pakistan^28^ highlights financial losses and a low-cost–benefit ratio for cotton growers. A yield gap of 57% exists between the average and potential yield of cotton in Pakistan, with the average yield being 53% lower compared to international standards^37^. Factors such as farmers’ low educational levels, improper fertilizer application, inadequate adoption of plant protection measures^38^, lack of technical knowledge, insufficient resources^39^, non-adoption of Integrated Pest Management (IPM) approaches^40^, pests attacks, excessive use of adulterated and expensive pesticides^41,42^, growing water scarcity^12^ limited access to extension services and weak linkages between research and extension^43^ directly and indirectly, contribute to the decline in cotton production. Another study by Anwar, Chaudhry^44^ highlight issues such as the unavailability of quality cotton seeds, insufficient water supply, and expensive inputs for cotton growers. Considering the prices of cotton, competing crops, and input costs, policy considerations could potentially enhance cotton yields^45^. However, in Pakistan, competing crops receive more support and better marketing prices, leading cotton growers to shift towards alternative crops like Sugarcane^46^.

Based on the literature reviewed above, we hypothesized a need for an in-depth study to explore the experiences of cotton growers in Punjab province. The literature shows that various research studies have investigated the reasons for the low cotton production in Pakistan and Punjab provinces. However, these studies have been quantitative and local in scope, limiting their generalizability. The key findings of the quantitative study encouraged us to conduct a qualitative inquiry to bridge the existing methodological research gap and explore the problem in detail. Qualitative research helps understand human meaning and experience dimensions, contributing to policy-making by illuminating research participants’ subjective meanings, actions, and social contexts^47^. Morse^48^ believed that qualitative research is generalizable, as it ensures a comprehensive, complete, saturated theory that applies to similar situations, questions, and problems beyond the immediate group. Malterud^49^ and Smith^50^ have endorsed the theoretical generalizability of qualitative studies.

To address this gap, we planned a phenomenological study focusing on the firsthand experiences of cotton growers. Open-ended questions were used to delve into the grassroots-level issues and establish connections between the different causes of cotton decline. Moustakas’s traditional phenomenological guidelines were followed in this research study. Numerous phenomenology scholars argue that individuals derive significance from their surroundings primarily through personal encounters and experiences^51–53^. However, exploring individual experiences is a highly complex and challenging task^54^. Thus, we opted for Moustakas’s traditional phenomenological guidelines to explore the experiences and elucidate the theoretical generalizability of this phenomenological study, which is the first in the context of Pakistani cotton farmers. This pioneering study holds significant implications for researchers, policymakers, and the industry, offering valuable insights into reviving cotton in the core cotton zone.

## Methodology

### Research approach

This study was qualitative, and a phenomenology approach was employed. Qualitative research is a method for comprehending research that concentrates on the distinctive techniques used to study social or human issues within their specific contexts^55^. In this study, we followed qualitative methodology to develop descriptive themes considering the experience of cotton growers. Some quantitative research studies based on traditional methods emphasizing factors affecting cotton production have been conducted in the Pakistani context. Still, farmers’ experience of growing cotton has never been explored qualitatively. To bridge this research gap, we followed a phenomenology approach because it explores the experiences from the individual’s perspective of experienced phenomena^56^. Creswell and Poth^55^ state that phenomenology is chosen as a primary approach to uncovering the significance of human experiences in terms of specific phenomena or significant cumulative events. The phenomenology approach has previously been used in various areas (i.e.^57–59^ and is also viewed from diverse perspectives as the theory of philosophy, perception and methodology.

Phenomenology is divided into three primary types: existential, transcendental, and hermeneutical. According to Creswell and Poth^55^, Greening^60^ and Neubauer, Witkop^61^, transcendental phenomenology directs its attention to the fundamental essence of human experience. Existential phenomenology emphasizes examining real-life social situations and contexts, and hermeneutic phenomenology concentrates on the structure of textual data and the interpretation of language use. The key research issue in this study is the phenomenological capture of the lived experiences of cotton growers while growing cotton crops in the cotton zone. Therefore, our study is classified as a transcendental phenomenological inquiry exploring cotton growers’ lived experiences. This phenomenological study followed Moustakas’s traditional phenomenological guidelines. Moustakas’s phenomenological guidelines involve four steps: Bracketing, Intuiting, Analyzing, and Describing^60^.

### Study area

**Figure 2:**
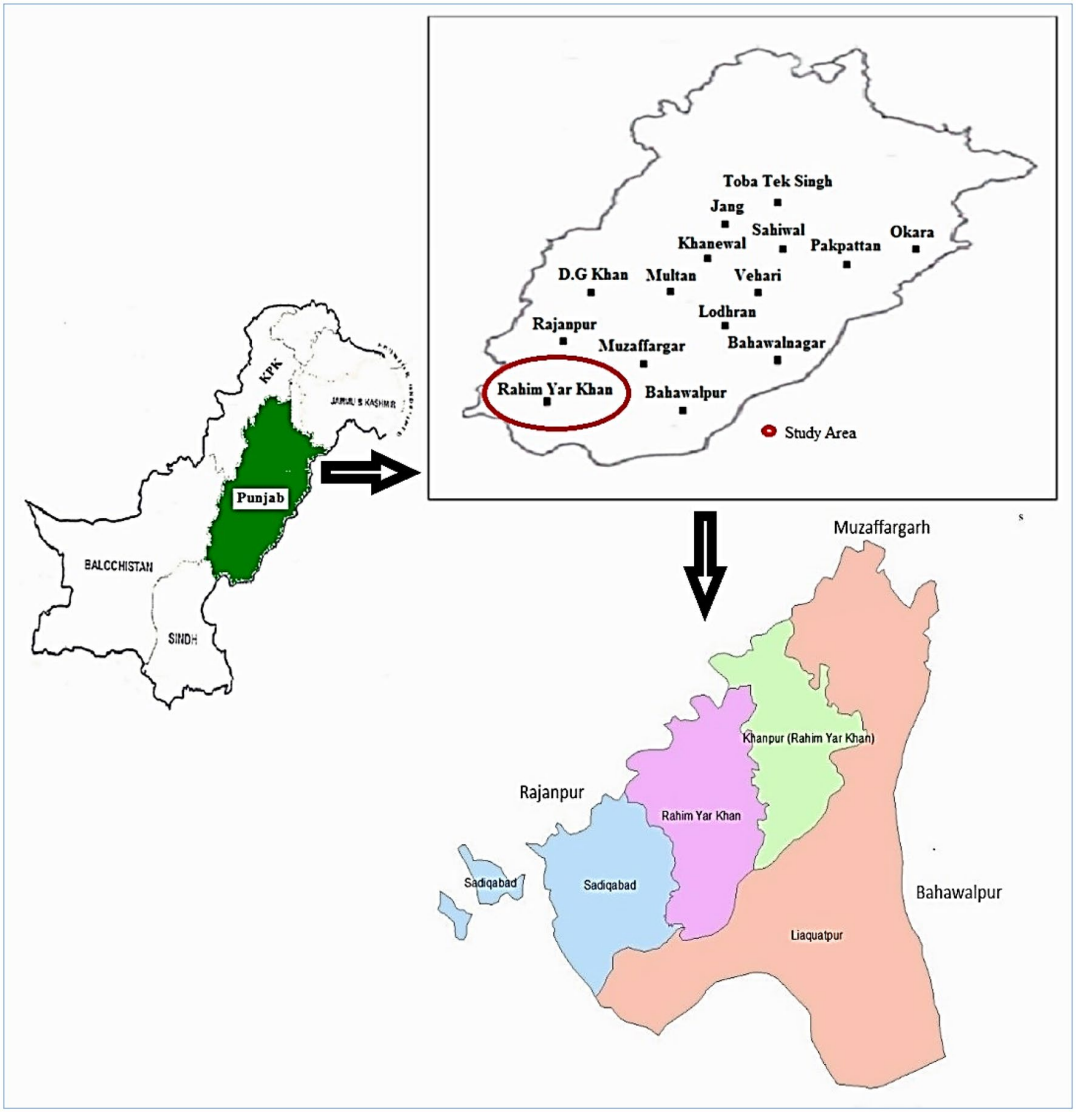
Map of study area, Pakistan, Punjab province and District.

Cotton is prominent in the Punjab province, particularly in the southern region, known as the core cotton zone. The Punjab has forty-one districts, further grouped into three major crop production zones viz northern, central and southern zones^62^. The south zone of Punjab is known as the core cotton-producing region because of intensive cotton cultivation (Fig. 2). The primary core cotton-producing districts are (i) Muzaffargarh, (ii) Multan, (iii) Bahawalnagar, (iv) Bahawalpur, (v) Vehari, (vi) Rahim Yar Khan (vii) Dera Ghazi Khan (viii) Layyah (ix) Sahiwal.

**Figure 3:**
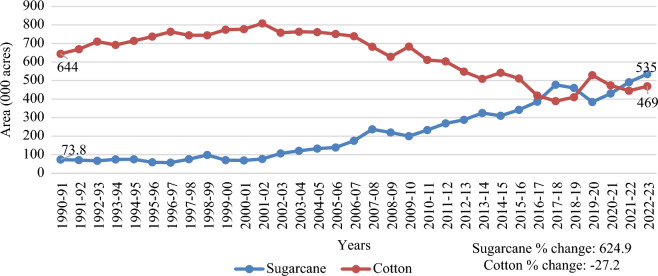
Area of cotton and Sugarcane from 1990 to 2023. Source^65^.

Of the core cotton zone, the district of Rahim Yar Khan in Punjab was chosen purposively as the research site due to its significant cotton production and abrupt change in cropping pattern from cotton to sugarcane crop. The area of sugarcane and cotton from 1990 to 2023 is described in Fig. 3, which shows a 624.9% increase in the sugarcane area and a 27.2% decrease in the cotton area. Rahim Yar Khan is the most suitable area for sugarcane cultivation because of its high sugar recovery^63^. Pertinent to these, six sugar mills are installed in this district out of 47 in the Province of Punjab^64^. The increased number of sugar mills is one of the key factors that motivated farmers to prefer sugarcane over cotton. The sugarcane area is expanding at the expense of the cotton area.

### Sampling procedure

Three specific criteria were employed to select participants for the study. Firstly, the informants had to be cotton growers. Secondly, they must possess a minimum of 10 years of experience in cotton cultivation. Lastly, the participants should have completed at least ten years of schooling. These criteria were established to ensure that educated and experienced cotton growers, capable of sharing valuable insights from their years of cultivating cotton, were included in the study. The study sample size was 10 cotton growers. The selection of study participants followed purposeful sampling, and the defined criteria served the intended purpose of the research. The goal of using a purposeful sampling approach was to form a sample encompassing a broader range of perspectives, thereby offering more comprehensive insights into the phenomenon under investigation, as emphasized by Creswell and Poth^55^. Maxwell^66^ further highlighted that the purposively selected interviewees provided specific details about the phenomenon being studied based on their experience.

In qualitative studies, saturation is the most commonly referenced justification for adequate sample size^48,67^. Different scholars have set different sample sizes that are adequate for generalizability. For instance, Constantinou, Georgiou^68^ suggested the lowest sample size for saturation 5 interviews. Guest, Bunce^69^ stated that data collection saturation can be achieved in 6–10 interviews. Similarly, Boyd^70^ suggested that data collection saturation is achieved in 2–10 respondents. Among scholars, Hennink and Kaiser^71^, based on a systematic review of 23 empirical studies, confirmed that saturation in qualitative studies can reach a relatively small sample size of 9–17 interviews. Large sample size in qualitative research was associated with wasting funds, leading to wasted data and overloading the respondents^72,73^. However, a sample size that is too small to reach saturation can decrease the validity. Based on the suggestions from different scholars and results of empirical studies, the sample size for this study was 10 respondents, although saturation was achieved more or less in 6–7 interviews. The population of this study was homogenous, and the sample could be a true representative of cotton growers in the region and those areas where cotton is deemed a preferred crop.

### Data collection and analysis

The data collection continued from June to July 2022, when the cotton crop was available in the field. Data were collected using an interview guide. Since the purpose of the study was an in-depth exploration of factors affecting cotton productivity, thus the interviews were kept open-ended. All ten interviews were conducted in Saraiki and Urdu, and the responses collected were converted into English. Each interview lasted, on average, 45–50 min, which was well in line with the guidelines presented by Smith, Flowers^74^ which stated that interviews should be confined between 45 and 60 min. Before the start of the interview, formal verbal consent was obtained from the study participants, and they were assured that their identity would be kept anonymous and that information would be used only for research. The interviews were recorded, and notes were also taken. Later on, a thematic analysis approach was used to analyze the collected data. Thematic analysis in qualitative studies focuses on identifying patterns, which are then reported as researcher-generated themes^75^.

### Ethical approval

The departmental committee first scrutinised this study and was notified via No. DFSS/260 Dated 17.02.2021. The professor heads this scrutiny committee, and the Associate professor and Assistant professor are members. In the next stage, this study was approved by the Directorate of Graduate Studies, which considered the university’s ethical considerations, guidelines, and regulations vide No DGS/26605-08 dated 05.08.2022. This study involved no experiment; instead, it was based on the survey, which the Departmental Scrutiny Committee and Directorate of Graduate Studies, University of Agriculture Faisalabad, approved. During the survey, formal informed consent was obtained from the respondents before the initiation of interviews. The respondents were assured in informed consent that their identity would be kept anonymous and the information they received would only be used for research purposes. Verbal consent was taken from the study participants and were ensured to keep their identity anonymous

### Findings

#### Data coding results

In-depth data were collected from the respondents. Around 527 statements related to the study’s objectives were identified in the data analysis process. Our team cross-checked all the identified statements and frequently created groupings of the meanings by reacting to research questions from the study participants. A total of 63 codes were generated from the statements. These codes were the terms repeatedly used by the participants. In addition, a total of 06 themes were organized from the clustered codes. The codes and themes are presented in Table 1.

**Table 1:** Clustered codes originated from the interviews.

Codes	Respondents										Total
	1	2	3	4	5	6	7	8	9	10	
Erratic rains	3	2	3	5	2	2	1	2	2	3	25
High temperature	1	1	0	1	1	2	1	2	1	1	11
Humidity	2	0	0	2	3	1	1	1	1	2	12
Moisture	1	0	0	0	1	1	0	0	0	0	3
Heat waves	2	1	1	1	0	0	1	0	0	1	7
Environmental pollution	2	1	1	0	1	1	1	1	1	0	9
Deforestation	0	0	0	1	0	0	1	1	2	0	5
Excessive nitrogen use	3	1	1	1	2	1	1	3	2	1	16
Flower shedding	2	3	1	1	1	1	1	1	3	4	18
Impure seed	5	4	4	3	5	6	3	3	3	4	40
Susceptible seeds	2	2	3	2	1	2	4	4	2	3	25
Poor germination	1	4	4	3	2	2	2	2	3	1	24
Mixing of seed	1	1	1	1	2	1	3	2	1	3	16
Seed monopoly	3	5	2	2	3	2	1	4	2	1	23
Unregistered varieties	2	3	3	2	4	4	6	1	2	3	30
Poor quality seed	3	1	4	2	4	2	1	2	3	1	23
Traditional varieties	1	1	1	1	2	2	1	2	2	0	13
Mistrust on varieties	2	4	4	3	2	1	1	3	3	1	24
Insects’ infestation	2	3	4	2	2	2	4	4	3	2	28
Pests outbreak	5	6	6	3	5	6	4	3	3	4	45
Diseases development	1	0	0	2	2	2	0	2	2	0	11
Virus	0	2	1	1	2	2	1	2	2	0	13
Fungus	1	1	0	1	2	2	2	3	2	0	14
Whitefly	8	6	6	4	5	6	4	4	3	5	51
Pink bollworm	3	4	4	3	2	3	4	4	3	2	32
Jasid	2	1	0	1	1	2	2	3	2	0	14
Thrips	1	2	0	1	1	1	2	3	2	0	13
Substandard pesticides	5	3	3	2	2	2	4	4	5	2	32
Wrong recommendations	3	5	4	3	5	6	4	2	3	4	39
Poor spraying techniques	4	3	4	3	2	4	3	2	5	3	33
Outdated chemistry	0	3	1	2	1	2	1	3	3	1	17
High cost of pesticides	5	4	3	4	5	2	2	4	3	2	34
Poor results	2	2	1	3	4	3	4	3	3	4	29
Excessive sprays	3	3	2	3	2	3	4	3	3	2	28
Improper use of nozzle	1	3	2	1	2	2	2	3	2	1	19
Health hazards	1	2	1	1	1	1	2	3	2	3	17
Poor role EFS	3	3	2	3	2	4	3	2	3	2	27
Sales oriented private EFS	4	5	3	2	4	3	4	5	4	3	37
Poor linkages	1	3	3	2	2	2	1	2	3	0	19
Biased services	1	2	3	1	2	1	3	2	4	3	22
No policy	3	5	2	3	3	2	2	4	2	2	28
Outdated research	2	2	3	2	4	4	3	1	2	1	24
No guidance on innovations	3	1	3	2	2	2	1	2	3	2	21
Unawareness	1	2	1	1	2	2	1	2	2	3	17
Poor adoption of recommendations	3	4	4	3	3	1	3	3	3	2	29
Risky crop	3	2	4	5	2	3	1	3	4	3	30
High cost of production	5	5	3	4	5	4	4	4	3	3	40
Inflation	7	5	4	3	4	5	4	3	5	4	44
Subsidies	3	4	2	3	2	3	4	3	3	2	29
Poor marketing	2	3	2	2	2	2	1	3	3	1	21
Cost–benefit ratio	1	2	1	3	1	1	2	3	2	3	19
Seed mafia	4	4	4	4	5	3	3	4	3	2	36
Pesticide mafia	3	5	3	3	4	3	4	3	4	5	37
Land rents	3	1	2	2	2	3	2	3	1	2	21
Black marketing	4	3	2	1	3	2	2	3	2	2	24
Sowing time	2	4	1	2	1	3	2	3	4	3	25
Imbalance use of pesticides	3	4	3	5	4	3	4	3	3	5	37
Imbalance use of fertilizers	2	3	3	2	2	3	1	3	3	2	24
Excessive nitrogen use	1	2	2	3	2	1	2	3	2	3	21
Cropping pattern	0	0	2	1	1	1	0	1	0	1	7
Labor issues	3	4	3	3	4	3	4	3	4	2	33
Mono cropping	1	0	1	1	1	0	0	1	1	0	6
Preventive sprays	2	3	2	2	1	2	2	1	2	2	19

#### Overview of the themes

The codes were rechecked, and a relationship was established between them. Thus, a total of six themes were grouped. These themes included (i) climate change, (ii) varietal problems, (iii) pesticide usage, (iv) sense of institutional services, (v) the attitude of farmers and (vi) soil health and environment.

**Table:** 

Theme		Sub-theme
1.	Climate change	Rainfall and humidity
		Fluctuation in temperature
2.	Varietal issues in cotton	Seed availability, quality and germination
		Seed marketing mechanism
3.	Pesticides use	Cost and quality of pesticides
		Application techniques
4.	Sense of institutional services	Polarized advisory services
5.	Attitude of farmers	Non-adoption of recommendation
		Inflation, subsidies and production cost
		Market failure
6.	Soil health and environment	Soil degradation
		imbalance use of inputs

### Theme 1: Climate change

Cotton is regarded as a risky crop and remains vulnerable to climatic changes. Study participants were all in agreement that climate change is the crucial factor determining the production of cotton. A favourable climate may bring a multifold increase in production. However, the recent surges in climate have destroyed the cotton crop. The production was badly destroyed despite the high production cost. Eventually, most farmers preferred to change their cropping pattern to other less risky crops like sugarcane. Climate-induced factors promoted insect pests and disease infestations, and the severity of pests’ attacks was unmanageable despite a multifold increase in the number of sprays. Farmers agreed that climate change had ruined the landscape of cotton crops for us.

#### Sub-theme: Rainfall and humidity

Almost all the study participants indicate that rainfall patterns have changed, and the intensity of the rainfall has increased in recent years. The increase in rainfall intensity was perceived as highly detrimental to the cotton crop as it invoked the attack of insects and pests. Respondents agreed that after the rainfall, the attack of Jassid increased, which reduced the plant growth, curled the leaves downwards and turned the leaves yellowish. As a result, the crop sheds its flowering, which causes a heavy loss in production. One of the respondents stated that;

For the last 3–4 years, the erratic rains ruined our crop, followed by the severity of the attack of Jassid and the white fly. Eventually, I faced a massive loss in the production of cotton.

Another farmer informed that; erratic rains increased the height of cotton because we usually apply more Nitrogen in the cotton crop. As a result, vegetative growth was fostered, and flowering remained short, causing a decline in production.

Respondents also informed us that rainfall-induced humidity and the population of Jassid and Thrips increased, causing severe damage to the plant. This implies that rainfall and humidity significantly affected the cotton crop.

#### Sub-theme: Fluctuation in temperature

Consistent and favourable temperature conditions are crucial for achieving optimal cotton yields. Fluctuations in temperature can disrupt the delicate balance of the cotton plant’s growth and development, leading to reduced productivity and economic losses for cotton growers, almost all the respondents agreed. Most cotton growers stated that sudden temperature changes increase the water stress on cotton crops, and farmers must change the irrigation interval. One of the respondents reported that:

The abrupt increase in temperature this year in June caused severe water stress, and I was compelled to squeeze the irrigation interval to four days to combat the water stress. In another case, this abrupt rise in temperature causes severe flower shedding.

Along with the water stress, the temperature rise also changed the population dynamics of the insects and pests of cotton crops. An increase in temperature led to heat stress, favouring the attack of particularly whiteflies, the most devastating pest of the cotton crop. It was concluded that weather was crucial for the cotton crop.

### Theme 2: Varietal issues in cotton

Respondents argued that quality seed is the pathway to the potential production of cotton. However, the foremost challenge we face while growing cotton is the seed. We do not get quality seed, even if we pay higher costs to get quality seed. The seed mafia in the country is responsible for destroying Pakistan’s cotton crop, as farmers agreed.

#### Sub-theme: Seed availability, quality and germination

The availability of quality cotton seed in the cotton-growing region is a matter of concern. We do not know where to go for the quality seed which could give us accurate germination. Respondents cited that getting seed from the Punjab Seed Corporation is difficult because of their limited offices and limited stock of varieties. Similarly, the germination of seeds from the Punjab Seed Corporation is usually poor. Alternatively, the private seed sector is a mafia, selling poor quality seeds with below-average seed germination. One of the experienced cotton growers said; we are deceived in the name of new varieties. Indeed, the varieties sold are of below-average quality and are unregistered.

During the discussion, one of the respondents shared his experience; I went to the dealer to purchase the Cotton variety SS102 because I had a good experience growing this variety last year. The dealer gave me SS1020 instead of SS102, saying this was a typing mistake on the bag. This was not SS102 because it grew poorly and produced more branches and fewer bolls.

Farmers shared their experience of poor seed germination and repeated seed sowing to fill the missing plants. This increased the cost and was a source of the decline in production.

#### Sub-theme: Seed marketing mechanism

Respondents stated that a proper marketing system for commercialising pure and certified cotton seed is lacking. The only government-led institution is Punjab Seed Corporation, which has limited offices, limited capacity, and limited dealers. Moreover, respondents were dissatisfied with the Punjab Seed Council’s quality and distribution system. One of the respondents stated that; I travelled 40 kms to get the Seed from the Office of Punjab Seed; when I reached there, I learned that the stock is over. My time, energy, and resources were wasted because of the non-availability of such a mechanism. This could help me identify whether a certain variety of seeds is available in stock in Punjab Seed.

Respondents reported the dominance of Private Seed Companies, and most companies sell unregistered seeds with poor germination. Respondents argued that more or less each pesticide dealer sells cotton seed, and surprisingly, most of them do not have seed-selling licenses. There is no mechanism for fixing seed prices. Each company and each dealer has its own rate. Respondent admitted a significant difference in the seed prices between Punjab Seed and Private Seed companies. The availability of many varieties, variations in rates, and many seed-selling shops have become problematic for the farmers, who must choose which variety and shop for cotton seed. Each dealer deceives the farmers in the name of a new variety, high-yielding potential and more than 80% germination. However, the matter of concern is how the unapproved seed is being sold in the market and how those varieties come out of the research stations. Cotton growers had delved into mistrust due to this price and seed quality difference.

### Theme 3: Pesticides use

As a risky crop, the respondents considered pesticide application inevitable to protect the crop from pest attack. Pertinent to weather patterns, susceptibility of varieties, and irresponsive pesticides, the application of pesticides on cotton crops has increased significantly. Respondents agreed that cotton intake ranges from pesticides, weedicides, insecticides, and fungicides to sowing and picking.

#### Sub-theme: Cost and quality of pesticides

Respondents were more concerned with the cost and quality of the pesticides. Respondents showed extensive concerns about the hefty increase in some sprays on 1 acre of cotton crop, which increased the cost of production and dented profits. Almost all the respondents had a mutual consensus that s. Farmers are being manipulated from both ends. On the one hand, the cost of pesticides is very high, and on the other hand, the results of the pesticides are poor. More importantly, farmers have to repeat the sprays, encountering the fear of crop yield loss by investing a lot of money in pesticides. The respondent during the discussion reported that;

This is true that the number of sprays has unexpectedly increased, and due to adulterated pesticides, I faced a serious decline in yield in the last 2–3 years. The pesticides purchased, especially from generic companies, were a total loss and even caused stress to the crop. Since then, I have preferred better-quality multinational groups than generic ones.

Regarding quality, all of the respondents preferred multinational groups over generic ones. However, products either from multinational or generic groups are expensive. One grower stated his experience;

I was sitting in a pesticide shop somewhere when a small farmer entered and asked for the products against the control of the high population pressure of Aphids. I was surprised when the pesticide dealer recommended spraying Imidacloprid, a very weak chemical, against the high population pressure of Aphids on cotton. I am sure the dealer suggested the chemical without logic and merely for his sale.

This was a notion that wrong recommendations and selling obsolete chemicals were the driving factors behind the increase in several sprays. Respondents were informed that the increase in sprays and the application of weak chemicals created resistance in insects and pests. It can be observed that cotton growers are compelled to spray 6–10 sprays against whiteflies only. The availability of substandard and obsolete products in the market is profitable for the dealers, but the cost and resources are intensive for the farmers. This situation becomes more severe when the Field Officers of the pesticides companies having matriculation and minimal experience in farming. They are targeted only for selling the product. They make the farmer believe that pesticides are the only source that can help you increase crop production. Respondents also believed that non-technical field staff is the reason for increasing pesticide applications.

#### Sub-theme: Application techniques

We explored that the spraying technique and the quality of the pesticides were of great worth. Unfortunately, all respondents had agreed on the farm’s adoption of improper spraying techniques. It was revealed that two modes of pesticide sprays are practised in the cotton area. First, getting the crop sprayed by hired labour (spray men) is more prevalent, especially among small growers, which are large in number. Second, spray can be applied using a mounted spray tank and a boom sprayer, which large farmers usually adopt. The use of modern tools like Sprays through Agricultural Drones was negligible among respondents for different reasons. Farmers argued that we mostly hire labour to spray. These labourers charge around 60–70 Rupees/25 Liter Tank, and 120–140 Liter of water is often used in 1 acre of cotton crop. These spray men are untrained and are practising spray based on their experiences. Due to the unavailability of trained labour and skilled spray men, small farmers are bound to ask them to spray. They use traditional nozzles, which have no other required nozzles, and more often, their machine has fixed nozzles that cannot be changed. Being unskilled, the chances of pesticide air drift remain higher, which further contributes to environmental degradation and is a source of health hazards to farm labour. Respondents had inclusive thoughts that pesticides being marketed are irresponsive and improper spraying techniques are used, which end in developing resistance in insects and pests. The respondent specifically cited that;

Aphids attacked the cotton crop in my field. I sprayed 2 sprays of Clothianidin and Thiamethoxam, which are some important chemicals but did not get any control on the Aphid. The quality of pesticides that we are using can be estimated from here.

Respondents pointed more seriously to the marketing of substandard quality pesticides, unregistered pesticides, and obsolete chemistries, which have no result on insect pests and diseases. There was a mutual consensus among respondents that inappropriate spraying techniques are major plights towards insect pests’ resistance, pesticide drift, environmental degradation and health hazards.

### Theme 4: Sense of institutional services

The role of institutions like research, extension, industry and academia is deemed to achieve high yield through the adoption of recommended production practices and technical guidance. The participants of this study were dissatisfied with the role of different institutions. Respondents argued that institutions fail to address the needs of the farming communities.

#### Sub-theme: Polarized advisory services

The study participants believed that institutional roles especially concerning the cotton sector, are polarized, where farmers are not their priority. The work of institutions is biased towards progressive farmers, and the needs of the small farmers remain unfelt. There is a clear divide between the public sector extension and the private sector extension. First, the staff of public sector extension rarely visit the farmers. The private extension sector, such as pesticides, focuses on their sales targets. They trigger their efforts not for the farmers but for their sales targets and incentives. One of the growers argued that;

If the private sector favours the farmers, then estimate how many companies have the latest chemistry, which could have given them significant control over insect pests and diseases. The majority of the generic companies are selling obsolete chemistries.

A few more respondents stated that the public sector extension field staff also recommends the same obsolete chemistries. Respondents argued that we have engaged in cotton cultivation for around 15–20 years and observed that the quality of pesticides and fertilizers decreases each year. Meanwhile, the availability of unregistered contaminated products in the market is increasing. It is well known that low-quality fertilisers have been marketed for the last couple of years and are being prepared at home by elite people. Black marketing of fertilizer has increased. This implies that the problem is becoming even more severe despite the institutions’ efforts. However, the sufferings of the farmers are increasing because he is compelled to purchase inputs at black rates. In case of delay, his crop may fail to grow. This situation points to institutional failure. One study participant responded that;

None of the sectors is properly working to make the farmers aware of the new technology. The system of guidance has been replaced with sale tactics. Public sector field staff rarely visit the farmers; the private sector focuses on sales.

Similar thoughts were presented by another farmer, who said there was a time when public sector extension was very active and used to pay visits frequently. Still, I do not know why they have limited themselves to their offices.

The respondents perceived that they were somehow convinced of public sector extension staff because of their guidance, which mainly focused on farmers’ development rather than manipulating farmers to meet their sales targets. During the discussion, respondents cited the recommendation of public sector extension field staff for customized biopesticide application on cotton. However, this biopesticide did not show promising results on a large scale.

Study participants agreed that institutional linkages are poor, and eventually, the benefits do not reach the farmers. Single institutions cannot meet the farmers’ needs. Research needs to consider the needs of the farmers. Farmers highlighted that the climate is changing, and climate-resistant cotton varieties are much needed. However, we still do not have accessible varieties that can be said to be heat resistant. Farmers showed their awareness about the CKC (triple gene) varieties of cotton, which are famous for being glyphosate-resistant and Pink Boll Worm Resistant. Farmers were partially satisfied that due to CKC, only 50% of the chances of Pink decreased, as we still have only 50% of the chances of Pink decreasing, and we still had to spray twice against the Pink Boll Worm. Respondents urged during the discussion the regulation on sustainable use of resistant varieties to improve their shelf life.

### Theme 5: Attitude of farmers

Study participants stated that the attitude of cotton growers was of great importance to the success of cotton production. Respondents believed that farmer attitudes do have a role in cotton failure. Stereotypic behaviour of farmers, non-following the guidance of experts and adopting the conventional approaches could be significantly associated with cotton crop failure.

#### Sub-theme: Non-adoption of recommendation

Farmers endorsed the non-adoption of recommendations among many cotton growers, which are pathways to getting high yields and bringing sustainability to the soil, environment and ecosystem. Adulterated pesticides, non-availability of fertilizers, and high rates of fire and electricity are the key challenges farmers face, and they are unable to adopt numerous recommendations. Respondents admitted that farmer adoption behaviour depends on many factors, including socio-economic, technological, psychological, and institutional factors. The key factor affecting the adoption was the farmers’ socio-economic conditions. The farmer makes a decision considering his economic condition. During the discussion, one respondent stated that;

When farmers have a financial deficiency, they try to access credit inputs. Most farmers get credit from the middleman, especially on an interest base, and he also picks the produce from farmers. Eventually, in the end, farmers get no benefit.

It can be seen that over the years, the economic condition of the farmers has worsened, and due to this, the adoption of recommendations is widely seen as poor. Another respondent said that farmers prefer using pesticide products from multinational companies when they have money in their pockets. When they do not have money, they get pesticides from generic companies on credit. Buying products on a credit basis usually enables dealers to market substandard inputs. This can be deduced that adoption is directly associated with strengthening the farmers’ socio-economic conditions. Moreover, technical support and guidance from the institutions could augment the socio-economic conditions of the farmers. One of the respondents stated that;

Farmers have no mistake because a large majority of farmers in Pakistan are less educated, captured in poverty, and have poor socio-economic conditions. As a result, farmers continue to adhere to the conventional approach of farming lazy adopters of the latest technologies, which are beyond their affordability.

Due to a lack of finance, farmers often use those fertilizers which are less costly. This was attributed to the lack of finance, as farmers were more inclined towards using Nitrogen (urea), significantly when the price of DAP exceeded 14,000 Pkr/bag (around 50$). As respondents revealed, buying two bags of DAP for one acre of cotton for a small farmer was impossible.

#### Sub-theme: Inflation, subsidies and production cost

During the discussion, respondents expressed their distress about the record inflation in the country, which has caused a multifold increase in the cost of production and curtailed the net profit share of farmers. Respondents agreed that the production cost has increased due to inflation and black marketing. Meanwhile, the owners of the lands have increased the rent. Farmers reported that land rents for one acre have crossed around 1 lac, which is turning unaffordable. Further, the issue is exacerbated due to inflation and crashed marketing systems. Farmers believed they always saw profits and how much they would earn after the investment. Farmers do not take risks but rely on rates and profits. One of the respondents specifically cited that;

I am growing cotton but not getting the profits. Whereas, in my neighbourhood, a person cultivated Maize twice a year, obtained around 200 Monds of production, and sold at the rate of 3200 Pkr/40 kg, which was highly profitable compared to growing cotton. He eventually decided not to grow cotton in the future because he got benefits from the maize.

This implies that farmers seek benefits, but inflation and the high significantly impact production costs and farmers’ mindsets. This may lead them to go for alternate crops because farmers always decide to choose a crop considering the price. Respondents cited the availability of subsidies for small farmers in particular. The government announced many subsidies for fertilizers, seeds, and some selective weedicides. Respondents argued the initiation of direct marketing and increased support prices rather than announcing subsidies. Farmers believed subsidies did not reach the small farmers. One of the respondents reported that I had sent many vouchers to the government number, but I did not receive that amount. Meanwhile, illiterate small farmers are unaware of and unable to encounter such a rigorous process of availing subsidies. Therefore, they are deceived and trapped by the agents.

### Theme 6: Soil health and environment

Respondents had a mutual consensus that soil fertility has decreased over the years. Soil structure is not favouring the farmers as it was around a decade ago. That made the soil infertile. Farmers perceived different factors that made the soil infertile, such as climatic factors and excessive usage of inputs, followed by traditional soil management procedures.

#### Sub-theme: Soil degradation due to imbalance use of inputs

Respondents argued that farmers apply fertilizers and other inputs on a “guess” basis, ignoring the recommendations. Respondents agreed that most of us have never gotten our soil and water tested. A couple of respondents stated that soil and water testing facilities are inadequate. In contrast, the reports received from the Soil and Water Testing Laboratory are not satisfactory. One of the respondents cited that;

Some time ago, the representatives from the Public Sector Extension took the samples from my land and said that I would get the report very soon. And so far, after two years, I haven’t received that report.

Farmers’ behaviour towards soil and water testing was poor, and inputs were mainly used based on their experiences. Therefore, farmers reported excessive usage of Nitrogen in the soil. At the same time, the use of micronutrients is scanty. The mode of application of inputs is usually traditional. As a result, soil fertility is not sustainable. To get high yields, farmers have to add extensive inputs. Extensive pesticide applications also adversely impact soil properties, the environment, and human health. Respondents also narrated the adverse impacts of pesticides, especially on the health of labourers and spraymen, who usually do not follow safety measures.

Respondents do believe that monocropping was also the reason for soil degradation. Farmers followed the cotton-wheat cropping pattern, and replicating the same for many years made the soil exhaustive. In the context of fertiliser patterns, they used the same soil preparation and fertilizer pattern, and applying pesticides severely influenced the soil structure. Farmers reported that crop rotation and green manuring were not used during the discussion, which could have helped soil restoration. One of the respondents raised an exciting question that, with the emergence of Glyphosate Resistance Cotton Varieties, the use of weedicide “Glyphosate” which is a non-selective weedicide, has increased and will continuously increase as well in future. We are sure this might have adverse impacts on soil microbes. Thus, there is a need to streamline the research on the safe use of glyphosate in the soil.

## Discussion

Previously published studies are focused mainly on quantitative findings exploring the different factors affecting cotton production and ranking those constraints based on quantified responses. This limitation in studies allowed us to examine the diversified factors of cotton’s drastic decline deeply. As a result, this phenomenological study presents the root causes and interconnected factors contributing to the failure of cotton.

Theme 1 Climate change indicates that climatic variation is the leading factor affecting cotton production^76^. The effects of abiotic stresses on growth and development are more conspicuous than those of biotic stresses^77^. Weather and erratic changes in the climate were perceived as significant risks by farmers that had adverse impacts on the cotton yield. In a study, Amjad Bashir, Batool^78^ identified a significant association between humidity, rainfall and weather conditions with the development of the thrips population in cotton. Similarly, Basit, Farhan^79^ reported that temperature indirectly influences insects’ biology, such as sex ratio, survival, life span, fertility, and fecundity.

Meanwhile, temperature also affects distribution, colonization, life history, behaviour, and insect fitness^80^. Increasing temperatures accelerated the development of phenological stages, leading to a shortened duration of crop phenological phases^36^. This implies that temperature was associated with the development of insects and pests’ pressure on the cotton crop.

A comprehensive analysis was presented by Raza and Ahmad^33^ about the impact of climate change on cotton productivity in Pakistan. They believed that a temperature increase of 1^0^C at the time of sowing encouraged cotton yield by 1.65% and 6.57% in the case of Bt cotton and conventional cotton varieties. The rise in temperature by 1^0^C at the vegetative and flowering-fruiting stage reduced the cotton yield by 24.14% and 8% in Bt and traditional varieties. Whereas, an increase in temperature at the crop’s maturity and picking stage of cotton by 1^0^C increased the yield by 26.54% in the Punjab province. On-field observations and farmers’ overviews also explain that the climate has become perplexing from the last seven to eight years, and farmers cannot comprehend the severity of the environment. As a result, farmers have to face hefty losses in terms of crop yield decline and increasing cost of production. Another diaspora is that farmers do not have adequate options and adaptive capacity to cope with climate change. For instance, climate-resistant varieties and climate-smart agricultural practices are relatively inaccessible to farmers. Temperature variations directly impacted the cotton yield; if it increases in the flowering stage, it may result in production loss. Therefore, if available and adopted by the farmers, heat-resistant varieties can curtail yield loss. For every 1 °C increase in average temperature, cotton yield decreased by 1.64%^81^. Various studies such as del Río, Anjum Iqbal^82^, Khattak and Ali^83^ and Ali, Khattak^84^ have confirmed an increase in temperature in Pakistan and an increasing trend of high temperatures in coming years. In contrast, climatic events are projected to increase in the future^85^ followed by longitudinal variation in precipitation^84,86,87^. According to^34^, minimum temperature harmed cotton yield, and a study Jaffar Iqbal, Roman^88^ arbitrated that cotton production declined due to erratic rainfall frequency, rain intensity, and heat waves. Research conducted Luqman, Karim^89^ found that cotton growers perceived that climate change indicators like temperature and erratic rainfall hurt cotton yield. This implies that there could be more drastic impacts on cotton due to climate change in the coming years if the climate-resistant varieties are not developed and adopted on the farm level. Climate change is associated with a decrease in cotton yield; thus, it is indispensable to identify those traits which could resist climatic variations^19,20^.

The current study under theme 2 also found that along with climate change, poor quality seeds, susceptible varieties and marketing of costly unapproved varieties were the key constraints. Growers urged for genetically modified, high-yielding and climate-resistant varieties, as previously reported by Shahzad, Mubeen^19^. Research studies such as Ali, Chaudhary^30^, Arshad, Khan^90^ and Ashraf, Sangi^31^ have reported that cotton production was declining due to the non-availability of quality cotton seed, poor genetic diversity and existing of unstandardized procedures of variety approval. Consequently, the unapproved and poor-quality seeds are being marketed by the dealers in the cotton regions, which is not only lowering the yield but also creating stress on genetic diversity. Literature is also scanty on how farmers face the problem of accessing quality seed and how they perceive the role of public sector institutions, which are mainly concerned with seed marketing and ensuring the availability of quality cotton seed. Farmers often cultivate poor-quality seeds due to unawareness and availability of many unapproved varieties in the market. The seed marketing system in the province is very ordinary, as many dealers are involved in the marketing of seeds. Farmers are deceived in the name of the latest variety, high production potential and provision of seed based on credit facility. Dealers are not held accountable for marketing clones, unapproved and poor-quality seeds. This study also explored the inadequate role of different institutions as perceived by the farmers (reported in Theme 4).

Farmers were dissatisfied with the role of research, extension, academia and other concerned institutions. They believed departmental linkages were poor and benefits did not reach the farmers. The services of institutions are polarized. Theriault^91^ reported that cotton growers were dissatisfied with the role of agriculture organizations. As far as public sector extension was concerned, they were biased towards large farmers and had inadequate capacities, were poorly trained, had fractional support from the government, undoable targets, irrelevant duties, were dissatisfied with their job roles and confused working environment as reported by Ali and Safdar^92^, Ashraf, Hassan^43^, Ashraf and Yousaf Hassan^93^, and Davidson, Ahmad^94^. Various research studies have reported that private sector extension was more effective in serving the farmers as compared to the public sector due to the timely dissemination of information and effective technical support to farmers and the use of obsolete technologies by the public sector^35,95,96^. Research studies such as Talib, Ashraf^97^ and Riaz, Ashraf^98^ agreed that the public sector was perceived as more effective than the private sector. This debate is well in line with the results of the current study that for some farmers, the private sector was effective, and for others, the public sector was good. However, the private sector had more of an edge because of the input distribution. The public sector only gave farmers technical advice, but they had to go to the private sector for input. Pertinent to input distribution, the private sector was criticized for its focus on sales and targets and generating benefits^94,99,100^. According to a recent study conducted by Abedullah and Ali (2024), the informal sector plays a significant role as the primary seed supplier in Pakistan’s agriculture industry. Numerous unregistered entities operate within this sector, distributing seeds to farmers through intermediaries without adhering to proper labelling and branding practices.

Consequently, substandard seeds are circulated and utilized, contributing significantly to the low productivity observed in agriculture. Local companies often offer substantial profit margins (30% and higher) to incentivize their dealers, enticing them to promote seeds of usually inferior quality. Unlike multinational firms, local companies tend to provide their dealers with more lucrative profit margins^101^. This issue of subpar seed quality is further supported by the findings of Muhammad Habib-ur-Rahman, Ashfaq Ahmad^102^, which project an 8% decrease in seed cotton yield in Pakistan by 2039 and a 20% decline by 2069. While this trend may increase profits for firms and dealers, it also signifies a significant future decrease in cotton production.

Results of this study presented in Theme 3 are evident that pertinent to the sale and target motives of the pesticide companies followed by substandard quality of pesticides and wrong recommendations specially made by the dealers were contributing causes of the increase in the number of sprays on cotton crop. A study by Khan, Mahmood^103^ reported pesticide overuse by cotton growers, followed by Tariq, Afzal^104^, who found that the cotton growers overuse and misuse of pesticides. Cotton absorbs 80% of the total pesticide use in Pakistan, yet with a tiny increase in yield^105,106^. In a study, Khan, Naeem^106^ recommended that 5–6 sprays were enough to control insect pests in cotton crops, considering the economic threshold level. However, farmers are found applying more than sprays to control pests. During a discussion with participants, farmers reported spraying 18–20 sprays in 1 acre of the cotton crop. Pertinent to this, the cost of production increased significantly. Results are complemented by Freire^107^, who states that the cotton crop had the highest production price among all annual crops. This suggests that improvement in spraying techniques and efficiency is much needed to control spray drift, improve the control of pests, and curtail environmental pollution^108–110^. Results were also precise, indicating that cotton growers were using improper spraying techniques, causing pesticide loss, an increase in the cost of production, environmental pollution, and health hazards. Pests can be controlled more efficiently if the spraying techniques are improved by targeting the right rate, time, and equipment^111^. In a study, Khooharo, Memon^101^ reported that farmers had inadequate awareness of the spraying techniques.

In theme 5, pesticides and fertilizer were associated with the attitude of cotton growers, which is more often negative as farmers hardly adopt the recommendations for different reasons. In the context of pesticide use (elaborated under theme 4), cotton growers knew about the pesticides but the problem was the non-adoption of recommendations regarding pesticide application^112^. In another study, Tariq, Afzal^113^ identified that cotton growers were still adopting traditional practices, and around 8–13 sprays were used on cotton crops with short intervals and more than the recommended dose to control insect pests of cotton. Meanwhile, farmers used traditional nozzles for each spray. Despite this, the right type of nozzle, velocity and size of the droplet are of great worth in the effective control of insect pests and the sustainability of the environment^114^. It was reported by Ejaz, Akram^115^ that due to the non-adoption of recommended nozzles, around 50% of total pesticides sprayed are lost, having poor control of pests, adding up to the cost of production and degradation of the surrounding soil, water and air environment (as explored in theme 6). Cotton growers prefer readily available, easy to handle, and apply pesticides^116^. This conventional approach may be linked to the farmers’ attitude towards buying products on a credit base from dealers. In this facility, the dealer provides pesticides to the farmer, and the farmer returns the money after crop harvest.

Dealers more often charge high markup rates on pesticides, which is one of the contributing factors to the overall increased cost of production. Poor knowledge of pesticides and the harmful effects of pesticide exposure was reported by Ejaz, Akram^115^. Whereas, Lekei, Ngowi^117^, Ngowi, Mbise^118^, Karamidehkordi and Hashemi^119^ also endorsed those farmers had poor knowledge of using pesticides recommended dose due to their poor education and lack of training regarding pesticide management. Eventually, environmental issues are increasing over time, and resistance in insect pests is mounting as well. A recent study Hussain, Ghramh^120^ reported increasing infestation levels of PBW on cotton each passing year. Ali, Aslam^121^ found that cotton growers had a high cost of production of cotton, including land rent, input application, crop management, and seed. The increase in input cost lowered the overall return of farmers. Non-adopters of the best management practices increased production by 11% and reduced the cotton output by 15%^122^. Studies such as those^123^^124^ reported that an intensive quantity of pesticides is used on cotton crops, which also increases the external social cost because these chemicals create severe threats to the environment^125^. This was also confirmed by Lampridi, Sørensen^126^ that conventional modes of input application like pesticides and fertilizer had their role in the deterioration of the environment, land and water resources. This debate endorses the findings of the current study that farmers had more inclination towards imbalanced use of inputs and excessive application of pesticides followed by an increase in a number of sprays, which have serious repercussions for the environment. Thus, there is a great challenge for researchers, policy practitioners, governments and agricultural departments to increase the cotton production and initiate environmentally friendly strategies around the world.

## Conclusion and recommendations

The study presents comprehensive insights into the multifaceted challenges impacting cotton production in Pakistan. Climate Change emerges as a pivotal factor, revealing the pronounced influence of climate fluctuations on the viability of cotton farming, with shifting rainfall patterns and intensified rainfall linked to increased insect and pest pressures. Varietal Issues in Cotton underscore the indispensable role of quality seeds in maximizing cotton yields. Yet, farmers lament the scarcity and substandard quality of seeds, attributing this to the influence of the seed mafia. Farmers had to face escalating costs and diminishing efficacy of pesticides, exacerbated by improper application methods and emerging resistance among pests. Sense of institutional services highlights farmers’ dissatisfaction with the efficacy of support from research, extension, industry, and academia, citing inadequate guidance and disjointed advisory services. The private sector is more involved in the cotton production system by providing inputs, technical services and, somehow, the marketing of produce. Public sector institutions are weak in capacities, and their mandate is not unfolding professionally. Due to the inefficiency of public institutions, farmers have to sell their produce at low prices, face a monopoly of middlemen, sell their produce at low prices, face a monopoly of middlemen, and access adulterated inputs. Varietal development in Pakistan is sluggish, and cotton varieties are available due to the changing environment and resistance against the increasing number of pests. Economic constraints, inflation, and decreasing profitability are impeding farmers’ attitudes towards cotton crops and compelling them to choose alternative kharif crops like sugarcane over cotton. Declining soil fertility and the adverse consequences of indiscriminate pesticide use advocating for sustainable agricultural practices to mitigate environmental degradation. Promoting climate-resilient farming techniques, ensuring access to superior seed varieties, fostering judicious pesticide management practices, bolstering institutional support networks, and prioritizing soil health preservation emerge as critical avenues for enhancing the sustainability and prosperity of the cotton sector. By embracing these strategies, stakeholders can work towards fortifying the resilience of cotton farming and uplifting the livelihoods of cotton growers nationwide.

The primary constraint of this study is that this study was limited to one district of the core cotton region. However, this area was worth studying because Sugarcane as an alternative crop is the primary threat to cotton revival. The population was homogenous, and sample size adequacy was confirmed concerning the saturation point advised by numerous qualitative empirical studies. Therefore, a study to the existing literature, especially in bridging the methodological gap. The existing literature on cotton is mainly quantitative, and this qualitative study, which underpins extensive viewpoints of cotton growers, will add value to the literature. Detailed exploration is also the pathway and guiding source for those countries that are prominent in cotton cultivation.

Based on the key results, the following takeaways are suggested:

- Research should encourage the cultivation of heat and drought-resistant cotton varieties to cope with fluctuating temperatures and extreme weather events. Therefore, the development of heat-resistant and early-maturing varieties is much needed.
- Government should support research and extension services to develop and disseminate climate-smart agricultural practices tailored to local conditions. Research and adaptive research farms in the cotton region should initiate different trials considering the local conditions. There is a need to promote the adoption of climate-resilient farming practices, such as drip irrigation to mitigate the impact of changing rainfall patterns and water stress.
- Strengthening seed certification and quality control mechanisms to ensure farmers have access to high-quality, certified cotton seeds with reliable germination rates is much needed. Increase the availability of improved and climate-resistant cotton varieties through public and private sector collaboration, including public research institutions and private seed companies. Punjab Seed Corporation needs to be strengthened.
- Enhance farmer awareness and training programs on integrated pest management (IPM) practices to reduce pesticide dependence and promote eco-friendly alternatives. Strengthen regulations and monitoring of pesticide sales to ensure farmers have access to registered and effective products with proper labelling and usage guidelines.
- Conduct farmer training programs to educate them about the benefits of adopting recommended practices and modern technologies, emphasizing long-term sustainability and increased profitability. Promote farmer participatory research and learning platforms to involve them in decision-making, allowing for feedback on research and extension activities. The support prices should be announced before the sowing starts. Moreover, there is a need to establish a mechanism for purchasing cotton on a government level.
- Collaborate with relevant stakeholders, including government agencies, research institutions, NGOs, and private sectors, to develop and implement comprehensive policies to improve cotton production and the livelihoods of cotton farmers. Raise awareness among farmers about the adverse effects of indiscriminate pesticide use on the environment and human health, promoting the adoption of safer and more targeted application methods.
- There is a growing need to formulate a Cotton Policy for cotton growers. This policy should engage all the concerned stakeholders with a strict accountability mechanism. There is a need for legal initiatives and their implementation against those selling adulterated seeds, irresponsible pesticides, polluting the environment and manipulating the market.
- Last but not least, this study is a framework for all stakeholders to invest and collaborate on the researchable issues for improving the cotton sector globally. Improvement of the cotton sector is the pathway for employment generation, industrial expansions and economic acceleration.

## Data Availability

We collected a bulk of responses during interviews. The datasets used and/or analysed during the current study are available from the corresponding author upon reasonable request.
